# Book Reviews

**Published:** 1904-09

**Authors:** 


### BOOK REVIEWS.

Proceedings of the American Medico-Psychological Association at the fifty-ninth annual meeting held at Washington, D. C., May 12-15, 1903. C. B. Burr, M. D., Secretary. Published by the Association. 1903.

Since the association whose annual volume of transactions is now being considered is at once the oldest and largest of the special medical societies, it is only reasonable to expect that it should contain a large mass of excellent material. Among such a group of interesting papers it is impossible to review adequately any one, much less all, but Stephen Smith presented one that will attract attention not less from the historical value it possesses, than the scientific interest it generates. The summary of Dr. Smith's paper we present below does imperfect justice to the original, which should be read to be appreciated.

The trial of William Freeman, a negro, for the murder of the Van Ness family near Auburn, was a cause célèbre in the administration of criminal justice in the middle of the last century. The crime was committed March 12, 1846, when, without notice or apparent motive Freeman, lately discharged from state's prison, killed John G. Van Ness, Mrs. Van Ness, an infant, Mrs. Van Ness's mother, and as then believed mortally wounded a laboring man who dwelt in the house,—a maid servant only escaping. The murderer was arrested, confessed the deed, but showed no signs of regret; indeed, he laughed during the recital of the incidents of the murder, betraying unmistakable signs of mental aberration.

The excitement was intense; the people revolted against the probable plea of insanity that might be made to secure acquittal of the prisoner, causing the sheriff to adopt vigorous means to protect him from the vengeance of the crowds at the jail, threatening to lynch the criminal. Hon. William H. Seward, who had already served two terms as governor and was destined to a larger fame, volunteered to defend Freeman, after becoming convinced from a careful study of the case that he was hopelessly demented.

Governor Seward had but lately secured the acquittal of a prisoner charged with murder, on the plea of insanity, and this only served to increase the feeling against a possible similar result in the present case, making the self-imposed task of Mr. Seward all the more difficult.

When the case was brought to trial the district attorney of Cayuga County, realising Mr. Seward's powerful influence and great ability, invoked the aid of the Attorney-General, Hon. John Van Buren, in conducting the prosecution. Mr. Van Buren, son of former president Martin Van Buren, was at the height of his popularity both as a lawyer and politician. He was familiarly known as "Prince John," and was a forensic and campaign orator of force and skill. The court-room was crowded to the doors and the two eminent counsel added interest to the proceedings, which had become almost dramatic in character.

The theory of the defense, as may be presumed, was insanity,—a plea that at the period of this trial had not reached the stage of abuse. Dr. Amariah Brigham, superintendent of the asylum for the insane at Utica, then the most famous alienist in America, was summoned for the prisoner. He had seen the prisoner once before the trial and unqualifiedly pronounced him mentally irresponsible. The application of counsel for the defense to permit Dr. Brigham to further examine and study the prisoner's condition was denied, a circumstance that served to accentuate the strained conditions that were arising. Dr. Brigham's testimony being regarded as the determining evidence, all eyes turned to him as he entered the witness box. He is described as a tall, spare man, with smooth, expressionless face, piercing eyes, and with a mien and bearing indicating intellectual superiority, with perfect self-control. He described, in response to Mr. Seward's questions, the symptoms of dementia at length, and gave with great clearness his reasons for determining that this special form of insanity afflicted the defendant. In the course of the direct examination he astonished the court and spectators by asseverating that he could diagnosticate insanity at sight, having often determined, he said, its absence when feigned, by simple observation without asking a question.

Attorney-General Van Buren, an adroit cross examiner, now took possession of the witness and addressed himself to the destruction of the splendid line of mental fortifications set up by Dr. Brigham, seeking by quibble and stratagem to neutralise the effect of the testimony he had given. Literature, science, history and philosophy, one and all, were invoked by turns to aid in the possible discovery of some weak point where a breach might be made in the armature of the witness, but in vain; on the other hand the learned counsel was frequently hoist with his own petard, so ready was Dr. Brigham in parrying his thrusts, often to the discomforture of the great lawyer. We cannot illustrate this phase of the trial better than to quote from the record:

"Is suicide contagious?" asked the counsel.

"I think it was in the French army until Napoleon put a stop to it," the witness replied. It is stated that a titter ran through the audience and the Attorney-General renewed the charge.

"Is hysterics contagious?" he asked.

"It seems to be catching," the doctor placidly said.

"Suppose, doctor," said the counsel, with a sneer, "that I should go out and steal a hundred dollars and then come in again and sit down here, would you swear I was insane?"

"I think I should," calmly replied the doctor.

The examination soon, however, assumed a more professional character, and Mr. Van Buren began to search into the details of the methods by which the witness proposed to diagnosticate insanity at sight. In answer to a question Dr. Brigham stated that he relied largely upon the features of a patient, which he studied closely.

"Which feature do you rely on in your diagnosis?" queried the counsel.

"I rely on no one feature, but study them as a group," was the answer.

"Do you rely on the chin," he was asked. "No," he said. "Do you you rely on the nose?" was the next question. "No," he said, "Do you rely on the ear?" the counsel persisted. "No," said the witness. "Do you rely on the cheek?" was the next tantalising question. "No," was the answer. "Do you rely on the mouth?" the counsel continued. "Very much," said the doctor. "Do you rely on the eyes?" was the next question. "Still more than on the mouth," the witness answered. "If, then, this prisoner were concealed all but his mouth or his eyes, you affirm that you could decide accurately whether or not he is insane?" queried Mr. Van Buren. "No, I do not state that; I must see all of the features at once," the witness urged. For a considerable time the astute Attorney-General dwelt on the features of different persons, endeavoring by his wit and sarcasm to throw the utmost ridicule upon the witness's method of detecting insanity at sight. With passive countenance and in the most quiet, self-possessed manner the witness answered all the questions, exhibiting not the slightest irritation at the gibes and jeers of the wily and witty counsel, as he held up to ridicule before the jury Doctor Brigham's new method of diagnosing insanity.

It is needless to say that by this time the interest of all concerned in the trial—court, jury, counsel,—as well as the great audience had reached a high pitch of nervous tension that was painful to endure and difficult to repress. The climax finally came however, when the Attorney-General, with impetuous heat and startling emphasis, demanded of the witness: "What! do you affirm that you can diagnosticate insanity at sight?" "I do," was the firm and dignified reply. Striding toward the witness, with thumbs thrust into the arm-holes of his waistcoat (a fashion much in vogue in those days) the great lawyer, turning toward the jury and spectators with a grandiose air commanded the learned doctor to "Point out to the court and jury an insane person in the audience." Now came the critical test of the competency of the witness, and he accepted the challenge without hesitation, confident in his ability to triumph.

Thus far we have given an abstract of Dr. Smith's graphic account, with the exception of two or three paragraphs quoted in full. Now, we prefer to let the distinguished narrator, who was present, describe the scene that followed in his own words:

A breathless silence fell upon the court room. The venerable judge raised his glasses to his forehead and surveyed the excited mass of people about to undergo the ordeal of an examination as to their sanity. The large number of legal gentlemen within the bar arose to their feet and gazed at the crowded hall and passageways with intense curiosity. The spectators were simply awe-stricken when they realised that the crucial test was to be applied to them, and, being one of the number, I still feel the thrill of horror I experienced.

Doctor Brigham arose from his chair very deliberately and stood for a moment surveying the people, as if to determine where to begin his scrutiny. He was as white and emotionless as a marble statue. Turning slowly to the left or first tier of seats he began a deliberate survey of the spectators, scanning the features of each one with the apparent confidence that he could detect the faintest traces of insanity. As his keen, searching eyes glanced from tier to tier of seats the suspense was simply unendurable. He had reached the middle aisle and yet no one had been pointed out as insane. Five hundred faces had been scrutinized and no group of individual features had responded to the test. That portion of the audience at least breathed more freely. An incredulous smile began to play about the mouth and light up the mobile features of the Attorney-General while a greater earnestness of manner and intensity of scrutiny were apparent in the witness. Deep furrows appeared on his pallid face, and his eyes assumed a piercing brilliancy which made every one shrink on whom his gaze was momentarily fixed. I felt myself transfixed when I realised that my face was focalised on his vision, and I experienced a sense of the greatest relief when I saw that I had safely passed the trying ordeal. A sigh of relief followed along the rows of seats as the glance of the great expert swept over them. The area of faces still to be examined was now rapidly diminishing, and but one-fourth of the audience remained to be scanned. It was apparent that thus far either there was no insane person in the crowd, or if there was, the witness had failed to detect such person, and hence had failed to answer the practical test to which he had been challenged by the prosecution and which he had accepted without protest.

Suddenly the wandering eyes of the expert became fixed; his features relaxed and assumed their customary impassiveness, and it was evident that he had discovered the object of his search. Stretching out his long arm and pointing with his finger toward a person on one of the rear tiers of seats, he quietly said, "There is an insane man." At the instant a man, as if struck with a bullet, sprang from his seat and, wildly gesticulating and shouting a volley of oaths against any one who would call him insane, rushed down the aisle toward the bar. The judge rose hastily from his chair as if about to escape, the lawyers were panic-stricken and mingled with the crowd; but Doctor Brigham stood perfectly self-possessed, while the officers struggled with the lunatic in their efforts to remove him from the court-room.

The whole scene was intensely dramatic and the termination was a surprising ovation for the triumphant actor, Doctor Brigham. The prosecution was completely nonplused, and the witness was allowed to retire without further tests of his ability as an authority in the diagnosis of insanity at sight. The man who was pointed out as insane proved to be a harmless lunatic who had strayed into court from a neighboring livery stable. To break the force of Doctor Brigham's successful test, however, the prosecution circulated the report that Mr. Seward, in anticipation of this test being made, had caused the insane man to be placed in that seat, and that Dr. Brigham had previously seen him. This absurd story only heightened the effect of the favorable impression which Doctor Brigham's successful answer of the challenge of the Attorney-General made upon the court, jury, and the people.

The final issue of the case was the conviction of the criminal for murder in the first degree. Public feeling would admit of no other verdict. He was not executed, but died in prison, demented to idiocy. An autopsy confirmed the correctness of the defense—insanity.

We have given much space to this narrative because, first, it relates to an important historic cause that involved scientific facts, almost forgotten at the present day; and, second, it will, we trust, prove of interest to the younger portion of our readers who are not acquainted with the medico-legal points developed in this celebrated trial; and, finally, because it cannot but prove interesting reading to all who may be called as experts to the witness stand.

---

American Edition of Nothnagel's Practice. Diseases of the Intestines and Peritoneum. By Dr. Hermann Nothnagel, of Vienna. Edited, with additions, by Humphrey D. Rolleston, M. D., F. R. C. P., Physician to Saint George's Hospital, London, England. Octavo, 1032 pages, fully illustrated. Philadelphia, New York, London: W. B. Saunders & Company. 1904. (Cloth, $5.00 net; half-morocco, $6.00 net.)

American physicians are not slow to take advantage of the best foreign literature, even though the productions of their own countrymen are of the best. The name of Nothnagel has been associated always since his earliest contributions, with the best medical literature and passes among physicians as genuine coin-current of recognised value.

Diseases of the intestines and peritoneum have possessed increased interest since surgery opened the way to the accurate diagnosis and successful treatment of many maladies formerly regarded as hopeless. It may be said, in general, that this treatise considers with intelligence and skill every known disorder that may affect the intestinal tract or peritoneum with an accuracy of detail that commends it to those who practise internal medicine, whether as family advisers or as consultants. The name of Humphrey D. Rolleston, which is attached as editor, is a guaranty of completeness from the English standpoint, while that of Alfred Stengel, as American editor, completes a trio of distinguished names that speak for the best work in the three principal countries of the world.

The editorial additions include sections on intestinal sand, sprue, ulcerative colitis, and idiopathic dilatation of the colon. Appendicitis and peritonitis have been given unusual space, treatment and diagnosis receiving exhaustive consideration. The section on intussusception has been greatly enlarged by the invaluable additions of D'Arcy Power, of England, who has made this subject particularly his own. There are twenty inserts of great merit.

A System of Practical Surgery. By Prof. E. von Bergman, M. D., Berlin, Prof. P. von Bruns, M. D., Tübingen, and Prof. J. von Mikulicz, M. D., Breslau. Volume II. Surgery of the Neck, Thorax and Spinal Column. Translated and edited by William T. Bull, M. D., Professor of Surgery, College of Physicians and Surgeons, New York, and Carlton P. Flint, M. D., Instructor in Minor Surgery. Royal 8vo., pp. 820. Illustrated. Lea Brothers & Co., New York and Philadelphia. 1904. (Price: cloth, $6.00; leather, $7.00; half morocco, $8.50, net.)

The successful issue of a new work on surgery is an achievement deserving of praise. The prompt translation into Spanish, Italian and English bears testimony of the worth of this one. The second volume deals with the surgery of the neck, thorax, and spinal column, under three general divisions of each topic,—malformation, injuries and diseases.

Particular interest will attach to the sections relating to malformations, diseases, and injuries of the thorax, mammary glands, spinal cord and vertebral column. Of late injuries to the spine, even to the extent of involving the cord, have become in many instances amenable to surgical skill. Professor Henle has contributed the section on this topic, hence it contains not only the latest observations pertaining to it, but the most authoritative surgery as well,—two considerations that serve to justify the praise which the article is receiving. Indeed, these remarks may well be applied to the other sections of the work, which everywhere evinces the careful preparation of experience. This second volume complements the first and proves the usefulness and propriety of this comprehensive surgical treatise.

---

A Guide to the Clinical Examination of the Blood for Diagnostic Purposes. By Richard C. Cabot, M. D. Octavo, pp. 569. Illustrated. Fifth revised edition. New York: William Wood & Co. 1904. (Price, $3.50.)

It is almost three years since the fourth edition of this most useful diagnostic aid was presented to the profession. Hematology, like other topics that may claim kinship, has advanced as might be expected proportionately to the general science of medicine, and Cabot, as might be expected of so scientific an investigator, has kept abreast of this progress. He has made changes in this edition to meet the introduction of the "Romanowsky" staining method, as applied to the routine of blood examinations. The colored plates, illustrating this method, are artistic exhibitions of both the method of examination of the blood and the color lithographic engraving.

The additions to the chapters on infectious diseases and blood parasites are such as to keep Cabot's work abreast of the progress-making in this field, and to keep the reputation of the book up to the standard it established for itself in the beginning,—the best.

---

A Practical Treatise on Medical Diagnosis for Students and Practitioners. By John H. Musser, M. D., Professor of Clinical Medicine in the University of Pennsylvania. Fifth edition, revised and enlarged. Octavo, pp. 1213; 395 engravings and 63 colored plates. Philadelphia and New York: Lea Bros. & Co. 1904. (Price, cloth, $6.50; leather, $7.50; half morocco, $8.00 net.)

From the first Musser's Medical Diagnosis has been an advocate of the clinical laboratory as an aid to accuracy, and the author enjoys the supreme satisfaction of seeing this method come into vogue both as to public and private practice.

The several sections of the book consider in their order of sequence, general considerations, including data, methods, objects, morbid processes and their symptomatology; historical, subjective, objective, physical, laboratory, and special diagnosis. The teaching of the book all the way through is precision; only by observing accuracy of observation and method can this be obtained; hence, Musser counsels the most untiring effort and indefatigable energy in the direction of precision.

This edition represents a thorough revision of the entire topic, and brings the various methods of diagnosis in all their details forward to the immediate present. It is by far the best illustrated treatise on diagnosis excepting, perhaps, Glentworth. R. Butler's, and is quite the largest yet issued. In spite of an attempt to keep the size of the book within the limits of former editions, so much of importance has developed in this field of late that Musser has felt compelled, however reluctantly, to increase the size of the book, until now it contains over 1,200 pages. Yet, in the face of this fact it does not seem unwieldly because, in part, it is so interesting, and in other part it does not contain a verbose sentence or an unnecessary phrase.

It is about as necessary for student and physician to possess Musser's treatise on diagnosis as to have in his library a standard work on human anatomy.

---

A Reference Handbook of the Medical Sciences...Embracing the Entire Range of Scientific and Practical Medicine and Allied Science. By various writers. A new edition, completely revised and rewritten. Edited by Albert H. Buck, M. D., New York City. Nine volumes, imperial octavo. Volume VIII. Illustrated by chromolithographs and 435 half-tone and wood engravings. New York: William Wood & Co. 1904. (Price, muslin, $6.00 per volume; leather, $7.00 per volume; half morocco, $8.00 per volume.)

The final volume of this great work is a worthy companion of its predecessors. It embraces the titles from U M B to Z Y M inclusive. Included, of course, in this alphabetical limit is all the recent literatures on the urethra, urine and urinary bladder, uterus, vagina and vaccination. A most exhaustive contribution on the urine is from the pen of Alfred C. Croftan, Chicago; another, on the uterus is by Henry D. Beyers, Philadelphia; and still another on vaccination, is by Samuel W. Abbott, Boston. These three articles are well worth the price of the volume. About one third of this volume is given to an appendix containing the advances and improvements that have taken place since the other volumes were issued. A copious index completes this most excellent number and is a fitting finale to one of the most comprehensive and useful reference works that has ever been issued. The profession of medicine owes a debt of gratitude to the indefatigable editor, the accomplished Dr. Buck, and to the publishers, William Wood & Company,—to the one for his untiring labors in the interest of scientific medicine and the others for their willingness to engage in such an enterprise involving the investment and tying up of large capital before returns could come in.

International Clinics. A Quarterly of Illustrated Clinical Lectures and especially prepared original articles on Treatment, Medicine, Surgery, Neurology, Pediatrics, Obstetrics, Gynecology, Orthopedics, Pathology, Dermatology, Ophthalmology, Otology, Rhinology, Laryngology, Hygiene and other topics of interest to students and practitioners. By leading members of the medical profession throughout the world. Edited by A. O. J. Kelly, A. M., M. D., Philadelphia. Volume II. Fourteenth series. 1904. Philadelphia: J. B. Lippincott Company. Cloth, $2.00.)

This number contains nine articles on the diseases of warm climates; four articles on treatment; three articles on medicine; five articles on surgery; one article on pediatrics; and one on rhinology. Among the foreign contributors are James Cantlie, of Edinburgh; Andrew Duncan, of London; C. Jarvis, of Paris; and S. Kanellis, of Athens. The home contributors include Frank Billings, Charles Greene Cumston, Daniel Eisendrath, Eli H. Long, Miles F. Porter, and James Edwin Thompson. Many others of distinction have also contributed to the book.

It is a practical volume, well illustrated, and is worth considerably more than the small price placed upon it by the publishers.

